# Ultrasound‐assisted recirculation extraction as an innovative approach for obtaining bioactive compounds from grape pomace

**DOI:** 10.1002/jsfa.70925

**Published:** 2026-07-30

**Authors:** Jailton Ribeiro Dos Santos Junior, Luiz Carlos Corrêa‐Filho, Dayvison Mendes Moreira, Renata Valeriano Tonon, Lourdes Maria Correa Cabral

**Affiliations:** ^1^ Graduate Program in Food Science, Institute of Chemistry Federal University of Rio de Janeiro Rio de Janeiro Brazil; ^2^ Graduate Program in Food Science and Technology Federal Rural University of Rio de Janeiro Seropédica Brazil; ^3^ Embrapa Food Technology Rio de Janeiro Brazil

**Keywords:** recirculation, high intensity, reuse, validation, kinetics, mathematical model

## Abstract

**BACKGROUND:**

Grape pomace, a by‐product generated during winemaking, still retains bioactive compounds with potential applications in the food, pharmaceutical and nutraceutical sectors. This work investigated ultrasound‐assisted extraction (UAE) coupled with a recirculation system as a strategy to improve their recovery. The effects of pump speed (909–1864 min^−1^) and ultrasound power (80–360 W) on phenolic content, monomeric anthocyanins and antioxidant capacity were assessed. Extractions were performed using ethanol–water solution (500 mL L^−1^ ethanol) at a solid‐liquid ratio of 50 g L^−1^ and kinetic assays established 90 min as the optimal extraction time.

**RESULTS:**

Total phenolic content varied from 8.55 ± 0.15 to 14.04 ± 0.14 g gallic acid equivalent kg^−1^. Antioxidant capacity varied between 10.11–18.31 g Trolox kg^−1^ [2,2′‐azino‐bis(3‐ethylbenzothiazoline‐6‐sulfonic acid)] and 7.32–12.90 g Trolox kg^−1^ (2,2‐diphenyl‐1‐picrylhydrazyl), whereas monomeric anthocyanins varied from 0.149 ± 0.006 to 0.236 ± 0.002 g malvidin‐3‐*O*‐glucoside kg^−1^. Ultrasound power significantly enhanced extraction efficiency as a result of intensified cavitation, whereas pump speed showed a synergistic effect at high power. Optimal conditions were established at 1725 min^−1^ and 320 W. The optimized system achieved greater extraction yields compared to conventional extraction, along with improved antioxidant capacity. Major phenolic compounds identified included gallic, vanillic and protocatechuic acids, catechin, epicatechin, *p*‐coumaric acid, quercetin, and ellagic acid, with malvidin‐3‐*O*‐glucoside as the predominant anthocyanin.

**CONCLUSION:**

UAE combined with recirculation proved to be a suitable process with scale‐up potential, enhancing extraction kinetics and bioactive compound recovery, supporting its application in grape pomace valorization. © 2026 The Author(s). *Journal of the Science of Food and Agriculture* published by John Wiley & Sons Ltd on behalf of Society of Chemical Industry.

## INTRODUCTION

Grape production exceeds 70 million tons annually, with almost half destined for winemaking. This process generates significant amounts of residues, particularly grape pomace, which accounts for about 200 g kg^−1^ of the processed grapes.^1^ Produced during the pressing stage, this by‐product is mainly composed of skins, seeds and stems, and has been explored for uses including beverages, fertilizers, oils, colorants and biorefinery products.^2^

This potential is largely linked to its chemical composition. In addition to carbohydrates, dietary fiber and minerals, grape pomace retains several bioactive constituents, mainly phenolic compounds such as phenolic acids, flavonoids and tannins. Among these compounds, anthocyanins are especially important because they show antioxidant, antimicrobial and anti‐inflammatory activities, which have been associated with health‐promoting effects.^3^, ^4^

Traditional procedures used to recover these compounds typically depend on high solvent consumption, prolonged extraction and heating. Such conditions can compromise thermolabile compounds, increase costs and generate environmental concerns.^5^, ^6^ Consequently, the search for extraction technologies combining efficiency, sustainability, and scale‐up potential has become a priority. Non‐conventional techniques, including ultrasound‐assisted extraction (UAE), microwave‐assisted extraction, ohmic heating and supercritical fluid extraction, have emerged as promising alternatives, showing that these processes still require study.^7^, ^8^

UAE has attracted attention because of its operational simplicity and extraction efficiency. Its effect is mainly associated with acoustic cavitation, a phenomenon characterized by the generation and subsequent collapse of microbubbles in a liquid medium, creating intense local conditions that favor cell disruption, improve mass transfer and promote the migration of bioactive compounds.^9^, ^10^ In recent years, UAE has been extensively applied for the recovery of bioactive compounds from various plant materials, including fruits, leaves, seeds and agro‐industrial residues, demonstrating higher yields of phenolic and antioxidant compounds compared to conventional extraction methods.^11^, ^12^, ^13^

Despite these advantages, the use of UAE at industrial scale still faces technical limitations, mainly because of uneven ultrasonic energy distribution, localized heating and process non‐uniformity, which can affect both compound stability and extraction performance.^10^, ^14^, ^15^ In conventional batch or probe‐type systems, these limitations may make an increase in volume difficult at the same time as maintaining process efficiency. Therefore, extraction systems able to combine high efficiency with improved process homogeneity are still needed, enabling the recovery of bioactive compounds with greater safety, lower cost and scale‐up potential.^15^

In this context, the present study investigated an ultrasound‐assisted recirculation system for recovering bioactive compounds from grape pomace, focusing on the combined influence of ultrasonic power and pump speed. Kinetic assays were also carried out to describe the extraction profile over time and a central composite rotatable design (CCRD) was used for process optimization and validation. By combining ultrasound with recirculation, the proposed approach was designed to enhance extraction performance and extract quality, supporting its use as a scalable strategy for the valorization of agro‐industrial residues.

## MATERIALS AND METHODS

### Materials

Grape pomace from Syrah grapes (*Vitis vinifera* L.) was supplied by Vinícola Inconfidência (Petrópolis, Rio de Janeiro, Brazil). The collected material was dehydrated in a tray dryer at 60 °C for 24 h, following the protocol reported by Mazza *et al*.^16^ The dried pomace was ground in a Mill 3000 hammer mill (Treu & Cia Ltda, Rio de Janeiro, Brazil) to produce a uniform powder. This material was vacuum sealed in laminated pouches and stored at approximately 6 °C until analysis.

### UAE and conventional extraction

UAE was performed with a high‐intensity ultrasound processor (model UIP1000hdT; Hielscher Ultrasonics, Teltow, Germany), **c**onnected to a recirculation setup consisting of a circulating pump, an extraction cell and a BS2d18 sonotrode (Hielscher Ultrasonics, Teltow, Germany). The operating frequency was 20 kHz. For all assays, the solvent was aqueous ethanol (500 mL L^−1^), with a solid–liquid ratio of 50 g L^−1^, as supported by previous studies on grape matrices, which reported that these conditions favor solvent polarity and mass transfer conditions for phenolic compound extraction.^17^, ^18^

The conventional assay was conducted under magnetic stirring at 200 min^−1^ using a hydroethanolic solvent (500 mL L^−1^ ethanol) and a solid–liquid ratio of 50 g L^−1^. The procedure was maintained at room temperature (approximately 25 °C).

Following extraction, the samples were centrifuged at 2683 × *g* for 15 min, after which they were kept at −20 °C. Extraction kinetics were assessed across all experimental design conditions, with aliquots collected at 15, 45, 90, 120 and 180 min.

### Experimental design

A CCRD was used to establish the most appropriate extraction conditions, considering two independent variables: pump speed (909–1864 min^−1^) and ultrasound power (80–360 W). Each variable was evaluated at two factorial levels, with three replicates at the central point and two axial points, giving a total of 11 experimental runs. The experimental responses were adjusted to a second‐order polynomial equation to describe the relationship between the independent variables and the response variables, according to:

(1) $$Y_{I}=b_{0}+b_{1}X_{1}+b_{2}X_{2}+b_{11}X_{1}^{\;2}+b_{22}X_{2}^{\;2}+b_{12}X_{1}X_{2}$$

where *Y*_i_ represents the response variables (total phenolic compounds, monomeric anthocyanins and antioxidant capacity); *X*_1_ and *X*_2_ are the coded independent variables for ultrasound power (W) and pump speed (min ^−1^), respectively; *b*_0_ is the intercept; *b*₁ and *b*_2_ are the linear coefficients; *b*₁₁ and *b*_22_ are the quadratic coefficients; and and *b*₁_2_ is the interaction coefficient.

Analysis of variance (ANOVA) and the coefficients of determination (*R* ^2^ and adjusted *R* ^2^) were applied to verify the adequacy of the experimental data to the proposed model. Three‐dimensional response surface plots were constructed to show the effects of the independent variables. The extraction condition was selected based on the response variables that were significantly affected by the experimental factors through the desirability function.

### Validation of the mathematical model

Eight additional experiments were chosen at random and carried out to validate the mathematical model. Validation was based on the comparison between predicted (*P*) and observed experimental (*O*) values. The precision factor $\left(A_{f}\right)$ and bias factor $\left(B_{f}\right)$ were calculated according to Baranyi *et al*. using:

(2) $$B_{f}=10^{\left(\text{Σlog}\left(P/O\right)/n\right)}$$

(3) $$A_{f}=10^{\left(Ι\log\left(P/O\right)I/n\right)}$$

where P is the predicted value, O is the observed value and n is the number of observations. The precision factor $A_{f}$ indicates the model's predictive precision, whereas the bias factor $B_{f}$ indicates the agreement between predicted and observed values. Values of $A_{f}$ and $B_{f}$ close to 1.0 are considered ideal, with a maximum acceptable deviation of 0.10 for each response variable.^19^

### Total phenolic compounds

Total phenolic content (TPC) was measured using the Folin–Ciocalteu procedure, according to Singleton and Rossi^20^ with adaptations described by Georgé *et al*.^21^ Absorbance was recorded at 760 nm using a UV‐visible spectrophotometer (UV‐1800; Shimadzu, Kyoto, Japan), and values were reported as g gallic acid equivalent (GAE) kg^−1^ dry weight.

### Monomeric anthocyanins

Monomeric anthocyanin content (MA) was quantified by the pH differential method, according to Giusti and Wrolstad.^22^ Absorbance readings were taken at 520 and 700 nm using a UV‐visible spectrophotometer (WUV‐M51; Bel Engineering, Monza, Italy) and the results were expressed as g malvidin‐3‐*O*‐glucoside kg^−1^ dry grape pomace.

### Antioxidant capacity

Antioxidant capacity was assessed using two radical scavenging assays: ABTS radical cation [2,2′‐azino‐bis(3‐ethylbenzothiazoline‐6‐sulfonic acid)], following Re *et al*.,^23^ and the DPPH (2,2‐diphenyl‐1‐picrylhydrazyl) radical assay, following the procedure of Rufino *et al*.^24^ Absorbance readings were recorded at 734 nm for ABTS and 515 nm for DPPH using a UV‐visible spectrophotometer (V‐M5; Bel Engineering). The results were reported as g Trolox equivalents kg^−1^ dry grape pomace.

### Determination of phenolic acids and flavonoids by HPLC

Free phenolic acids and flavonoids were determined according to Nascimento *et al*.^25^ The filtered hydroethanolic extract was introduced into the HPLC system with a photodiode array (PDA) detector (Alliance e2695; Waters Corp., Milfoprd, MA,, USA). Separation was performed on a reversed‐phase C18 column (Kromasil, 150 × 4.6 mm, 5 μm; Nouryon, Amsterdam, The Netherlands) at 40 °C, with a mobile phase consisting of phosphoric acid solution (1.5 mL L^−1^) (A) and acetonitrile (B) under gradient elution at 1.0 mL min^−1^. PDA spectra were acquired from 200 to 600 nm, and quantification was performed at the maximum absorption wavelength of each compound (270, 290, 310, 325 and 370 nm). Results were reported as g kg^−1^ dried sample.

Anthocyanins were identified by HPLC‐diode array detection (DAD) according to Santiago *et al*.,^26^ with minor modifications. Chromatographic separation was achieved on reversed‐phase C18 column. The mobile phase consisted of an acidified aqueous phase combined with an organic phase, applied under gradient elution. Detection was carried out in the visible region, near 520 nm. Compounds were assigned by comparing retention times and UV‐visible spectra with analytical standards and literature data. Quantification was carried out from external calibration curves, and results were expressed as g kg^−1^ dry residue.

### Statistical analysis

All analyses were carried out in triplicate, and the results are expressed as the mean ± SD. Data were evaluated by ANOVA, followed by Tukey's post‐hoc test at a 5% significance level (*P* < 0.05). Pearson's correlation analysis was also applied. Statistical analyses and figures were generated using Statistica, version 10.0 (StatSoft, Tulsa, OK, USA).

## RESULTS AND DISCUSSION

### Extraction kinetics

Figure 1 shows the kinetic behavior determined for the four factorial trials of the experimental design, whereas the other trials followed a comparable trend. Overall, the measured responses increased as extraction proceeded.

**Figure 1:**
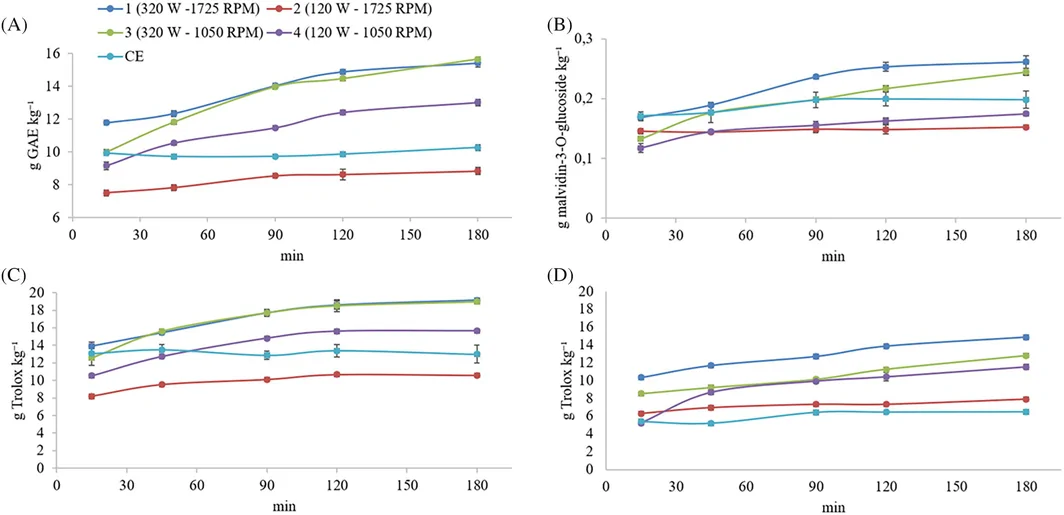
Evolution level of phenolic compounds, monomeric anthocyanins and antioxidant capacity throughout extraction of grape pomace. (A) Total phenolic compounds (TPC), (B) monomeric anthocyanins (MA) and (C, D) antioxidant capacity (ABTS and DPPH). 1, 2, 3 and 4 factorial experiments (Table 1).

The low deviations observed throughout the extraction kinetics suggest consistency in the analytical measurements and in the extraction system. Most response variables differed significantly (*P* < 0.05) over time, showing that extraction time influenced compound recovery. The only exception was observed for total phenolic content in treatment 1 (320 W and 1725 min^−1^), where the times of 90, 120 and 180 min did not differ statistically, indicating that the system probably reached equilibrium after 90 min. These findings reinforce the consistency of the observed kinetic pattern and show that the operating conditions affected compound release and diffusion.

Increasing the extraction time promoted a gradual increase in TPC, MA and antioxidant capacity (ABTS and DPPH). This trend is expected in solid‐liquid extraction, in which diffusion is favored by the concentration difference between the plant matrix and the solvent. However, after approximately 90 min of extraction, the curves began to level off, indicating that the system moved toward equilibrium. From this point onward, the increases in TPC, MA and antioxidant capacity were smaller, suggesting that most of the soluble compounds had already been extracted. This response agrees with the diffusion behavior described by Fick's second law, according to which, as the concentration gradient decreases, the mass transfer rate also declines, leading to a plateau in the extraction curve.^27^

This kinetic pattern has also been reported by Rocha and Noreña,^28^ as well as Goula *et al*.,^29^ which is consistent with the present results. It was previously observed that the extraction yield of bioactive compounds increases with extraction time, particularly during the initial stages of UAE, when cavitation enhances solvent penetration and cell disruption. As the process progresses, the system tends toward equilibrium and diffusion becomes slower. In this study, comparable behavior was observed, with extraction yield increasing up to 90 min and stabilizing thereafter.

To further support these observations, the kinetic behavior was compared between conventional extraction and the ultrasound‐assisted recirculation treatments presented in Fig. 1. Conventional extraction changed only slightly over time, reaching approximately 10.27 g GAE kg^−1^ after 180 min, indicating a diffusion‐controlled equilibrium. By contrast, ultrasound‐assisted treatments exhibited a continuous increase in TPC, MA and antioxidant capacity, particularly at higher ultrasonic power (320 W). Under these conditions, TPC reached approximately 14.48 g GAE kg^−1^ at 90 min and up to 15.81 g GAE kg^−1^ at 180 min. These results indicate that ultrasound improved both extraction efficiency and extraction kinetics compared to conventional extraction.

The greater extraction performance observed in the recirculation system can be attributed to the joint effect of acoustic cavitation and continuous solvent movement. Cavitation promotes mechanical effects, including microjet formation and disruption of plant tissues, which facilitates solvent entry into the matrix, whereas recirculation increases contact between the solvent and grape pomace particles, contributing to a more homogeneous extraction environment. Together, these effects favor solute migration from the solid matrix to the solvent and contribute to higher extraction yields.^10^, ^30^

Therefore, 90 min was chosen as the extraction time for the following assays, as this condition indicated near‐equilibrium between the solvent and the plant matrix. Increasing the process time to 180 min resulted in only minor gains (< 10%) in the concentration of bioactive compounds, which did not compensate for the higher ultrasonic energy demand. Moreover, longer ultrasound exposure may favor oxidative losses of phenolic compounds and anthocyanins, both of which are sensitive to heat and cavitation effects.^2^ Thus, 90 min was considered to provide a suitable balance between extraction efficiency, compound stability and energy feasibility.

Finally, the highest concentrations of bioactive compounds were observed in treatments 1 (320 W and 1725 min^−1^) and 3 (320 W and 1050 min^−1^), both performed at the highest ultrasonic power (320 W). This result indicates that increasing ultrasonic power enhances the recovery of bioactive compounds by intensifying cavitation, promoting greater cell disruption and improving mass transfer between the solid matrix and the solvent. This behavior is consistent with the findings of Drevelegka and Goula,^31^ who reported the positive influence of ultrasonic power on extraction efficiency.

### Total phenolic content, antioxidant capacity and anthocyanin extraction: effects of ultrasonic parameters and statistical correlations

TPC values were between 8.55 ± 0.15 and 14.04 ± 0.14 g GAE kg^−1^. Antioxidant capacity was between 10.11 ± 0.07 and 18.31 ± 0.10 g Trolox kg^−1^ for ABTS and between 7.32 ± 0.07 and 12.90 ± 0.07 g Trolox kg^−1^ for DPPH. MA values were between 0.149 ± 0.006 and 0.236 ± 0.002 g malvidin‐3‐*O*‐glucoside kg^−1^. Table 1 provides a summary of these results.

**Table 1:** Central composite design with coded and decoded values and experimental results.

T	Power (W)	Pump speed (min^−1^)	AC (g Trolox kg^−1^)			
			TPC (g GAE kg^−1^)	MA (g malvidin‐3‐*O*‐glucoside kg^−1^)	ABTS	DPPH
1	320 (+1)	1725 (+1)	14.04 ± 0.14	0.236 ± 0.002	17.68 ± 0.41	12.69 ± 0.13
2	120 (−1)	1725 (+1)	8.55 ± 0.15	0.149 ± 0.006	10.11 ± 0.07	7.32 ± 0.07
3	320 (+1)	1050 (−1)	13.97 ± 0.13	0.199 ± 0.004	17.72 ± 0.10	10.14 ± 0.00
4	120 (−1)	1050 (−1)	11.47 ± 0.10	0.156 ± 0.007	14.80 ± 0.07	9.92 ± 0.19
5	220 (0)	1387 (0)	12.92 ± 0.09	0.180 ± 0.014	15.47 ± 0.07	11.82 ± 0.18
6	220 (0)	1387 (0)	12.11 ± 0.15	0.171 ± 0.007	14.87 ± 0.27	10.97 ± 0.23
7	220 (0)	1387 (0)	10.76 ± 0.08	0.169 ± 0.012	14.71 ± 0.48	11.09 ± 0.15
8	360 (+α)	1387 (0)	13.36 ± 0.17	0.187 ± 0.007	18.31 ± 0.10	12.90 ± 0.07
9	80 (−α)	1387 (0)	9.10 ± 0.26	0.144 ± 0.001	12.26 ± 0.10	8.15 ± 0.15
10	220 (0)	1864 (+α)	10.78 ± 0.14	0.153 ± 0.002	13.83 ± 0.03	9.86 ± 0.06
11	220 (0)	909 (−α)	12.42 ± 0.03	0.161 ± 0.008	13.25 ± 0.16	11.54 ± 0.24

Abbreviations: T, treatments; TPC, total phenolic compounds; MA, monomeric anthocyanins; AC, antioxidant capacity (ABTS and DPPH).

The extraction data showed clear differences in total phenolic content, monomeric anthocyanins, and antioxidant capacity among treatments, indicating that both ultrasonic power and recirculation speed strongly affected extraction performance. These differences may be explained by the combined action of acoustic cavitation and fluid movement, which affect matrix breakdown and solute movement into the solvent during UAE.

Higher ultrasonic power generally resulted in greater phenolic compound recovery and antioxidant capacity, suggesting that greater cavitation intensity enhances cell wall disruption and facilitates compound release. This behavior is linked to stronger cavitation effects, which amplify mechanical effects such as microjet formation and shear forces, favoring disruption of the plant tissue.^9^

Similarly, variations in recirculation speed influenced the solvent–solid interaction, contributing to more homogeneous extraction conditions. In this context, pump speed affects the hydrodynamic conditions of the system, influencing the exposure of the material to the cavitation zone and the renewal of the solvent at the solid–liquid interface.

The highest TPC value (14.04 ± 0.14 g GAE kg^−1^), antioxidant activities (17.68 ± 0.41 g Trolox kg^−1^ for ABTS and 12.69 ± 0.13 g Trolox kg^−1^ for DPPH), and monomeric anthocyanins (0.149 ± 0.006 to 0.236 ± 0.002 g malvidin‐3‐*O*‐glucoside kg^−1^) were obtained under the combined effect of high ultrasonic power (320 W) and high recirculation speed (1725 min^−1^), indicating synergistic effects between these parameters.

Conversely, lower power or reduced recirculation led to decreased recovery because of insufficient cavitation intensity and less effective interaction between the solvent and the solid matrix. Overall, these findings confirm that the operational parameters of the ultrasound‐assisted recirculation system play a critical role in determining extraction efficiency and compound stability.

The TPC values obtained here (~8.50–14.00 g GAE kg^−1^) were comparable to those described for grape pomace but remained below values reported in other works.^16^, ^30^ This behavior may be related to different aspects, such as differences in raw material composition (grape variety, processing conditions and the extent of phenolic extraction during winemaking), as well as extraction parameters such as solvent composition, pH and solid–liquid ratio. It is important to highlight that the grape pomace used in this study was obtained after conventional red winemaking, where fermentation and pressing favor the migration of phenolic compounds to the must, leaving a solid residue with lower phenolic availability.^32^, ^33^ For example, Luque‐Alcaraz *et al*.^30^ reported similar TPC values (8.51–13.61 g GAE kg^−1^) using probe‐type ultrasonication, which is close to the values found here. By contrast, much higher values (64.85–117.32 g GAE kg^−1^) reported by Mazza *et al*.,^16^ despite using the same grape variety, may be explained by processing differences, particularly in sparkling wine production, where phenolic extraction is generally less intensive. Consequently, the composition of the resulting pomace differs significantly, which affects the amount of phenolics remaining in the pomace. Therefore, the observed differences indicate that extraction yield depends on both the characteristics of the raw material and the operational conditions employed.

When compared with conventional extraction,^34^ which yielded only 0.023 g Trolox kg^−1^ in antioxidant activity, the present method achieved markedly higher values, confirming the superiority of high‐intensity techniques. Although lower than those reported by Marianne *et al*.^8^ for Tempranillo pomace, the antioxidant capacities observed here showed a similar tendency, indicating that grape variety and processing conditions affect the results and confirming the positive relationship between phenolic content and antioxidant activity.

Similarly, regarding anthocyanin recovery, Rocha and Noreña,^28^ compared microwave and ultrasound assisted extractions of grape pomace and reported anthocyanin contents (1.05–1.20 g kg^−1^ malvidin‐3,5‐diglucoside) higher than those obtained in the present study. This difference is mainly attributed to the raw material juice pomace, which retains more anthocyanins than winemaking residues and to the use of higher ultrasonic powers. By contrast,^8^ reported lower anthocyanin levels (0.069 g cyanidin‐3‐glucoside kg^−1^) but higher antioxidant capacity, suggesting that their extracts contained larger amounts of other phenolics with strong reducing activity. Thus, extraction conditions and pomace characteristics help explain the variations observed among studies.

In addition to these comparative observations, the statistical analysis further demonstrated that the independent variables evaluated in this study had significant effects on TPC, antioxidant capacity (ABTS and DPPH) and monomeric anthocyanin content (Table 2). Ultrasonic power exhibited a positive linear effect on all responses, whereas it showed a negative quadratic effect on TPC. On the other hand, pump speed presented negative linear and quadratic effects on TPC but did not significantly affect antioxidant capacity or anthocyanin content when considered independently. However, the interaction between ultrasonic power and pump speed was significant: for TPC, this interaction contributed positively to extraction, as did the ABTS assay. These results indicate that the influence of ultrasonic power is predominant, especially in the extraction of monomeric anthocyanins, and that the combined adjustment of parameters can improve the recovery of bioactive compounds. From a mechanistic perspective, this predominance is associated with the direct role of ultrasonic energy in controlling cavitation intensity, which is the main driving force for cell disruption and compound release.

**Table 2:** Coded regression analysis and model fit for phenolic compounds, monomeric anthocyanins and antioxidant capacity

	TPC (g GAE kg^−1^)	AC (g Trolox kg^−1^)		MA (g malvidin‐3‐*O*‐glucoside kg^−1^)
		ABTS	DPPH	
β_0_ – intercession	1268.05*	60.32*	44.94*	18.41*
β_1_ – power (linear)	176.12*	9.56*	6.01*	3.27*
β_2_ – speed (linear)	−58.43*	1.06	−1.91	–
β_11_ – power (quadratic)	−64.75*	−1.95	−1.21	0.77
β_22_ – speed (quadratic)	−39.20*	−2.45*	−1.72	–
β_12_ – power × speed	74.80*	4.65*	5.14	1.11
Adjusted *R* ^2^	0.92	0.82	0.82	0.91
Lack of fit	0.10	0.05	0.50	0.87

* Significant at *P* < 0.05. TPC, total phenolic compounds; AC, antioxidant capacity (ABTS and DPPH); MA, monomeric anthocyanins.

The quadratic models fitted the experimental data well, as indicated by significant regression, adequate determination coefficients (adjusted *R* ^2^ ranging from 0.82 to 0.92), and lack of fit that was not significant (*P* > 0.05) (Table 2). These results indicate that the models were suitable to describe the responses under the conditions studied. However, for the monomeric anthocyanin response, the linear model fitted the data more appropriately, indicating that the axial points were not significant and were therefore excluded.

The modeling approach adopted in this work was deemed suitable for describing and predicting experimental data. Figure 2 shows the three‐dimensional response surfaces plots, illustrating the effects of pump speed and ultrasonic power on the evaluated responses.

**Figure 2:**
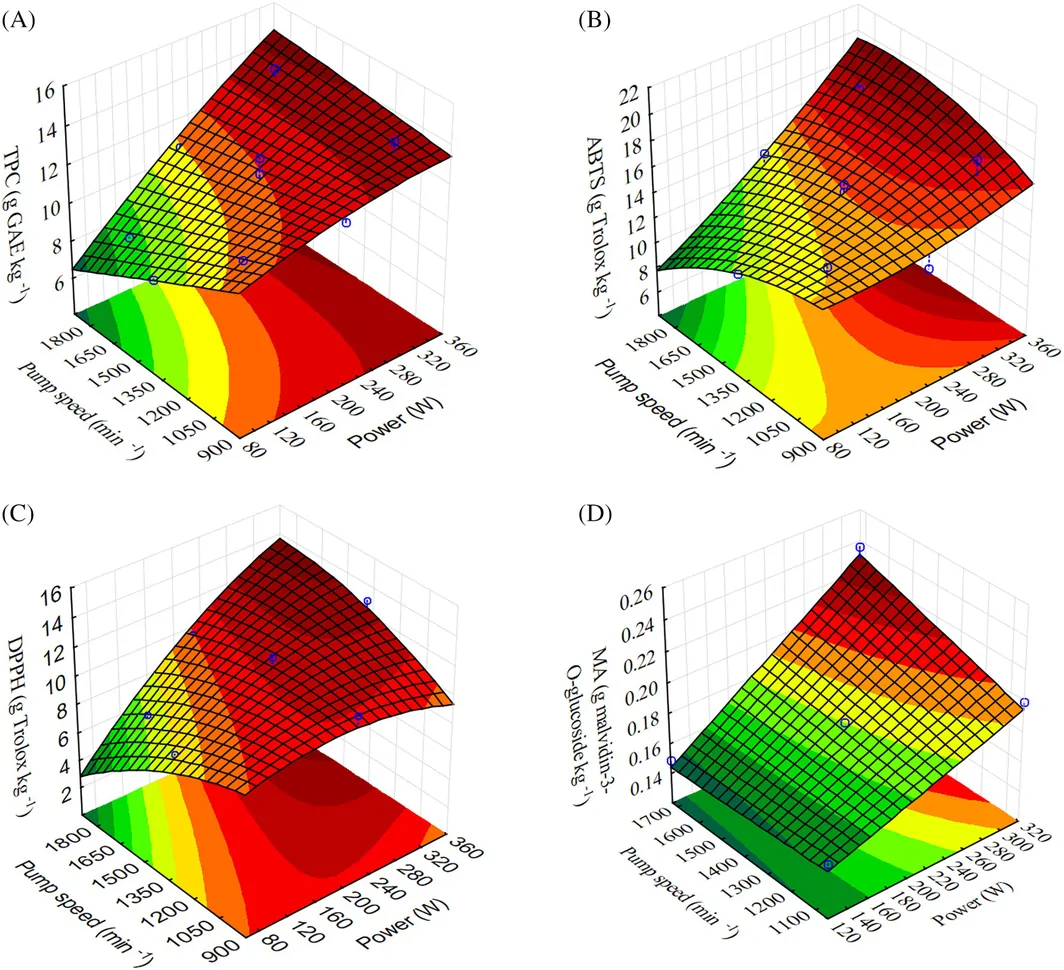
Response surface plots. Effect of speed and power on extracting total phenolic compounds (TPC), antioxidant capacity and grape pomace's monomeric anthocyanin (MA) content. (A) Total phenolic compounds (TPC), (B,C) antioxidant capacity (ABTS and DPPH) and (D) monomeric anthocyanins (MA).

Higher ultrasonic power increased TPC, antioxidant capacity, and monomeric anthocyanin content (Fig. 2). This response can be explained by stronger acoustic cavitation, in which the growth and collapse of microbubbles in the liquid medium produce shear forces and microjets that disrupt plant tissues and make the matrix more permeable.^11^, ^16^ The higher the ultrasonic power applied, the more intense the cavitation, which facilitates solvent penetration into the matrix and promotes compound release and overall extraction efficiency.^29^

The variability observed among studies employing UAE for bioactive compound recovery from grape pomace may result from several factors, such as solvent composition, temperature and amplitude, as well as intrinsic differences in the raw material, such as grape variety, cultivation conditions, maturity stage, processing and climatic characteristics.^35^, ^36^ Even with these differences, acoustic cavitation is recognized as a mechanical phenomenon that improves mass and energy transfer, favoring the release of bioactive compounds.^8^, ^30^

A higher ultrasonic power strengthens this effect, leading to increased extraction of phenolics and soluble pigments, which was also observed in the antioxidant responses (ABTS and DPPH), which followed the same behavior as TPC. This correlation indicates that the higher phenolic content directly contributes to the reducing capacity of the extracts, as also reported by Caldas *et al*.^18^ in grape skin extracts. In the case of anthocyanins, ultrasonic power stood out as the main determining factor, showing a positive linear effect, reinforcing the role of acoustic energy as a key variable in the extraction of pigments and phenolic compounds.

Pump speed also affected phenolic recovery and ABTS response, although its effect was weaker than that of ultrasonic power. This response may be linked to changes in the flow regime within the system, which may favor or reduce cavitation efficiency depending on the recirculation condition. At lower power levels (< 100 W), lower recirculation speeds (< 1000 min^−1^) favored extraction, probably because contact with the cavitation zone was improved. Conversely, higher speeds (> 1200 min^−1^) may lower extraction efficiency by reducing cavitation effectiveness. As the ultrasonic power increases, the effect of pump speed becomes less evident because stronger cavitation promotes cell disruption and compound diffusion regardless of the recirculation regime. Under these conditions, cavitation becomes the prevailing mechanism, reducing the relative impact of hydrodynamic factors on extraction performance. The interaction between power and pump speed showed a positive effect on TPC and antioxidant capacity (ABTS), highlighting the importance of optimizing these parameters for extraction efficiency.

In addition, the recirculation setup evaluated here provides advantages compared with batch extraction. Recirculation ensures better homogenization of the extraction medium, uniform distribution of acoustic energy and stability of power over time. These factors contribute to more uniform energy distribution and consistent exposure of the sample to cavitation, which are critical for process efficiency. It also allows operation with larger volumes and under conditions closer to real industrial applications, improving reproducibility and process control. Thus, although the isolated influence of pump speed is more evident at lower powers, it remains relevant to system dynamics and process performance, supporting the need for combined control of operating parameters.

Ultrasonic power and recirculation speed act together on extraction efficiency, modifying bioactive compound recovery and antioxidant response. This interaction shows the need to consider the statistical relationships and the underlying physical mechanisms involved in the process. Overall, extraction performance depends on the interplay between acoustic cavitation and fluid dynamics. Ultrasonic power controls the intensity of cavitation, promoting cell disruption and compound release, whereas recirculation affects solvent flow and contact with the solid matrix. An appropriate balance between these conditions is therefore necessary to maximize extraction efficiency and support the scale‐up and industrial application of the process.

To better examine the association among the responses, Pearson correlation coefficients were calculated to evaluate the relationships between TPC, antioxidant capacity and anthocyanin content, as summarized in Table 3.

**Table 3:** Pearson correlation coefficients among total phenolic content, monomeric anthocyanins, and antioxidant capacity (ABTS and DPPH).

Pair of Variables	Pearson (r)	t calculated	*P*‐value (two‐tailed)
TPC × MA	0.8200*	4.2980	0.0020
TPC × ABTS	0.9000*	6.1942	0.0002
TPC × DPPH	0.8400*	4.6444	0.0012
MA × ABTS	0.8100*	4.1437	0.0025
MA × DPPH	0.6900*	2.8599	0.0188
ABTS × DPPH	0.8000*	4.0000	0.0031

* Indicates significance (*P* < 0.05). TPC, total phenolic compounds; CA, antioxidant capacity (ABTS and DPPH); MA, monomeric anthocyanins.

Positive and significant correlations were observed for all variable pairs at the 5% level (*P* < 0.05). The results confirmed the strong relationship between phenolic content and antioxidant activity of the extracts. The highest correlation occurred between TPC and ABTS, suggesting that phenolic compounds had an important contribution to the antioxidant response, in agreement with previous reports. Studies by Caldas *et al*.^18^ and Mazza *et al*.^16^ reported that extracts with higher phenolic content exhibit stronger antioxidant activity in radical scavenging assays, such as ABTS and DPPH, due to the ability of these compounds to donate electrons or hydrogen atoms and neutralize free radicals.

The association between TPC and anthocyanins indicates that anthocyanins contributed importantly to the phenolic pool, highlighting their relevance among the bioactive constituents. Although the relationship between anthocyanins and TPC was high, it was slightly lower than that observed with ABTS, indicating that other phenolics, such as flavonoids and phenolic acids, also contribute significantly to antioxidant activity, as described by Llorent‐Martínez *et al*.^37^ The correlations between anthocyanins and antioxidant assays were more moderate, indicating that, although anthocyanins play an important role, the total antioxidant capacity depends on the synergistic action of a broader group of phenolic compounds. This behavior aligns with observations by Kashyap *et al*.^38^ who reported that cherry pomace extracts contain multiple bioactive compounds that are collectively responsible for their efficiency in free radical scavenging.

### Analysis of the extraction process

#### Desirability and validation of mathematical models

The desirability function was then used to determine the most suitable operating condition within the evaluated experimental design. A desirability value of 0.9 was obtained considering all response variables simultaneously. Desirability values above 0.7 are considered satisfactory.^39^ However, it is important to note that the desirability function is a mathematical optimization tool and does not by itself validate the scientific relevance or overall performance of the extraction process. Based on this criterion, the optimal extraction conditions were established at a pump speed of 1725 min^−1^ and an ultrasonic power of 320 W. The model was experimentally validated under these conditions, and the experimental results were compared with the values predicted by the desirability function. The predicted values were 13.67 g GAE kg^−1^ for TPC, 17.98 and 12.85 g Trolox kg^−1^ for antioxidant capacity (ABTS and DPPH, respectively), and 0.236 g malvidin‐3‐*O*‐glucoside kg^−1^ for MA. The experimental values obtained were 14.13 ± 0.58 g GAE kg^−1^ for TPC, 19.88 ± 1.94 and 12.27 ± 0.52 g Trolox kg^−1^ for antioxidant capacity (ABTS and DPPH, respectively), and 0.259 ± 0.009 g malvidin‐3‐*O*‐glucoside kg^−1^ for MA.

The experimental values were close to those predicted by the model, confirming that the selected condition was appropriate. These findings should be considered together with the experimental results and mechanistic discussion reported throughout the present study, which provide a broader evaluation of the extraction performance.

To further ensure the reliability of the developed mathematical models, quantitative validation was performed by calculating the precision and bias factors, as presented in Table 4.

**Table 4:** Validation of the mathematical models: precision and bias factors.

	TPC (g GAE kg^−1^)	AC (g Trolox kg^−1^)		MA (g malvidin‐3‐O‐glucoside kg^−1^)
		ABTS	DPPH	
$A_{f}$	1.06	1.06	1.06	1.06
$B_{f}$	0.97	0.95	0.97	0.98
MAD	0.03	0.03	0.03	0.02

Abbreviations: $A_{f}$, accuracy factor; $B_{f}$, Bias factor; MAD, mean absolute deviation; TPC, total phenolic compounds; AC, antioxidant capacity (ABTS and DPPH); MA, monomeric anthocyanins.

Model validation was performed using eight random experimental points within the CCRD, yielding $B_{f}$ and $A_{f}$ values ranging from 0.97 and 1.06. $A_{f}$ values indicate that the model possesses adequate predictive accuracy, whereas the $B_{f}$ values confirm its reliability. For monomeric anthocyanins, validation based on four additional randomized experimental conditions, resulted in $B_{f}$ and $A_{f}$ values between 0.98 and 1.06, confirming the precision and reliability of the model for predicting responses within the studied domain.

### Phenolic compound profile

The phenolic profile is shown in Table 5 for the extract obtained under the selected optimized condition. At the selected condition, gallic acid was the predominant phenolic compound in the grape pomace extract (0.2305 g kg^−1^), followed by vanillic acid (0.2215 g kg^−1^) and quercetin (0.1772 g kg^−1^). This profile agrees with previous reports describing phenolic acids and flavonoids as the main constituents of ultrasound‐assisted grape pomace extracts.^16^, ^18^

**Table 5:** Phenolic compound profile of grape pomace

Compound	Content (g kg^−1^)
Gallic acid	0.2305
Vanillic acid	0.2215
Quercetin	0.1772
Protocatechuic acid	0.0615
Catechin	0.0527
Epicatechin	0.0426
*p*‐Coumaric acid	0.0232
Ellagic acid	0.0148
Malvidin‐3‐*O*‐glucoside	0.0414
Peonidin‐3‐*O*‐glucoside	ND
Petunidin‐3‐*O*‐glucoside	ND
Cyanidin‐3‐*O*‐glucoside	ND
Delphinidin‐3‐*O*‐glucoside	ND

Abbreviation: ND, not detected.

Moderate levels of protocatechuic acid (0.0615 g kg^−1^), catechin (0.0527 g kg^−1^) and epicatechin (0.0426 g kg^−1^) were detected, whereas *p*‐coumaric and ellagic acids were present at lower concentrations, as commonly reported for winery by‐products.^40^, ^41^ The detection of gallic acid in the extract indicates that UAE may favor the release of phenolic compounds, including compounds associated with the cell wall matrix.^9^

Overall, the selected process recovered a phenolic fraction composed of different compounds with reported antioxidant and antimicrobial relevance, supporting the use of ultrasound‐assisted extraction with recirculation for grape pomace valorization.^42^ However, differences between analytical methods should also be considered. The Folin–Ciocalteu assay resulted in higher TPC values than the sum of individual phenolic compounds identified by HPLC. This difference is related to the low specificity of the Folin–Ciocalteu reaction, which measures overall reducing capacity and may also respond to interfering substances such as sugars and ascorbic acid, which can overestimate phenolic content.^43^ By contrast, HPLC selectively quantifies targeted compounds and may not detect polymeric, bound or unidentified phenolics. Therefore, the observed difference reflects methodological limitations and highlights the complementary nature of both analytical approaches.^44^

In the HPLC‐DAD analysis of anthocyanins, malvidin‐3‐*O*‐glucoside was the only compound detected and quantified, with a content of 0.0414 g kg^−1^ of grape pomace, whereas the remaining anthocyanins were not detected under the analytical conditions employed. This result may be related to the characteristics of the grape pomace evaluated in this study, since the residue came from conventional red winemaking. During fermentation, anthocyanins are extensively extracted into the must, leading to a significant depletion of these compounds in the residual solid matrix. In addition, anthocyanins are highly sensitive to environmental conditions such as pH, temperature and oxygen, and may undergo degradation or transformation into more stable or polymerized forms that are not easily detected by HPLC‐DAD.

This result agrees with previous reports, in which malvidin‐3‐*O*‐glucoside has often been described as the main anthocyanin in *V. vinifera* grapes, and its derivatives are relatively more stable during winemaking compared to less substituted analogues.^45^ Furthermore, pigment extraction during enological processes is selective, and more stable compounds tend to persist in grape pomace, whereas others are preferentially extracted or degraded.^46^ Together, these chemical and processing aspects help explain both the predominance of malvidin derivatives and the limited diversity of anthocyanins observed in the present study.

## CONCLUSIONS

UAE with recirculation proved to be an efficient approach for recovering bioactive compounds from grape pomace under the evaluated conditions, resulting in enhanced phenolic content and antioxidant capacity. These findings highlight its potential for improving extraction performance. Both ultrasonic power and recirculation speed significantly influenced extraction performance, particularly affecting phenolic and antioxidant contents. The optimized conditions (1725 min^−1^ and 320 W) yielded extracts rich in phenolics and anthocyanins, confirming the effectiveness of the extraction process.

Overall, the ultrasound‐assisted recirculation system represents a promising and scalable strategy for the valorization of agro‐industrial residues. Future studies should focus on process scale‐up, energy efficiency, and the stability and application potential of the obtained extracts in food and nutraceutical formulations.

## AUTHOR CONTRIBUTIONS

JRDS, LCC‐F and DMM were responsible for investigations. JRDS, LCC‐F and MLCC were responsible for formal analysis. JRDS was responsible for writing the original draft and data curation. LCC‐F, RVT and MLCC were responsible for methodology. LCC‐F, RVT and MLCC were responsible for review and editing. RVT was responsible for conceptualization. RVT and MLCC were responsible for visualization. MLCC was responsible for conceptualization, resources, supervision, project administration and funding acquisition.

## FUNDING

This study was supported by Fundação Carlos Chagas Filho de Amparo à Pesquisa do Estado do Rio de Janeiro (FAPERJ), Coordenação de Aperfeiçoamento de Pessoal de Nível Superior (CAPES) and Conselho Nacional de Desenvolvimento Científico e Tecnológico (CNPq).

## CONFLICTS OF INTEREST

The authors declare that they have no conflicts of interest.

## Data Availability

The data that support the findings of this study are available from the corresponding author upon reasonable request.

## References

[1] Castellanos‐Gallo L , Ballinas‐Casarrubias L , Espinoza‐Hicks JC , Hernández‐Ochoa LR , Muñoz‐Castellanos LN , Zermeño‐Ortega MR *et al*., Grape pomace valorization by extraction of phenolic polymeric pigments: a review. Processes 10:469 (2022).

[2] Ferreira‐Santos P , Nobre C , Rodrigues RM , Genisheva Z , Botelho C and Teixeira JA , Extraction of phenolic compounds from grape pomace using ohmic heating: chemical composition, bioactivity and bioaccessibility. Food Chem 436:137780 (2024). 10.1016/j.foodchem.2023.137780 37879228

[3] Chakka AK and Babu AS , Bioactive compounds of winery by‐products: extraction techniques and their potential health benefits. Appl Food Res 2:100058 (2022).

[4] Voss M , Gaudino EC , Tabasso S , Forte C and Cravotto G , Current emerging green technologies for the valorization of grape and cherry wastes. Curr Food Sci Technol Rep 1:47–61 (2023).

[5] Ivanovic J , Tadic V , Dimitrijevic S , Stamenic M , Petrovic S and Zizovic I , Antioxidant properties of the anthocyanin‐containing ultrasonic extract from blackberry cultivar ‘Čačanska Bestrna’. Ind Crop Prod 53:274–281 (2014).

[6] Machado APDF , Pasquel‐Reátegui JL , Barbero GF and Martínez J , Pressurized liquid extraction of bioactive compounds from blackberry (Rubus fruticosus L.) residues: a comparison with conventional methods. Food Res Int 77:675–683 (2015).

[7] Crescente G , Cascone G , Petruzziello A , Bouymajane A , Volpe MG , Russo GL *et al*., A comparative study between microwave hydrodiffusion and gravity (MHG) and ultrasound‐assisted extraction (UAE): chemical and biological characterization of polyphenol‐enriched extracts from Aglianico grape pomace. Foods 12:2678 (2023). 10.3390/foods12142678 PMC10378583 37509770

[8] Lizárraga‐Chaidez M , Abadía‐García L , Mendoza‐Sánchez MJ , Hernández‐Mendoza EL and Mendoza‐Sánchez M , Optimization of the green extraction process of antioxidants derived from grape pomace. Sustain Chem Pharm 37:101396 (2024).

[9] Chemat F , Rombaut N , Sicaire AG , Meullemiestre A , Fabiano‐Tixier AS and Abert‐Vian M , Ultrasound assisted extraction of food and natural products: mechanisms, techniques, combinations, protocols and applications. A review. Ultrason Sonochem 34:540–560 (2017). 10.1016/j.ultsonch.2016.06.035 27773280

[10] Yusoff IM , Mat Taher Z , Rahmat Z and Chua LS , A review of ultrasound‐assisted extraction for plant bioactive compounds: phenolics, flavonoids, thymols, saponins and proteins. Food Res Int 161:111839 (2022). 10.1016/j.foodres.2022.111268 35761580

[11] Al‐Dhabi NA , Ponmurugan K and Maran Jeganathan P , Development and validation of ultrasound‐assisted solid‐liquid extraction of phenolic compounds from waste spent coffee grounds. Ultrason Sonochem 34:206–213 (2017). 10.1016/j.ultsonch.2016.05.005 27773237

[12] Liao J , Guo Z and Yu G , Process intensification and kinetic studies of ultrasound‐assisted extraction of flavonoids from peanut shells. Ultrason Sonochem 76:105661 (2021). 10.1016/j.ultsonch.2021.105661 PMC8343109 34252684

[13] Xiang Y , Liu Z , Liu Y , Dong B , Yang C and Li H , Ultrasound‐assisted extraction, optimization, and purification of total flavonoids from Daphne genkwa and analysis of their antioxidant, anti‐inflammatory, and analgesic activities. Ultrason Sonochem 111:107107 (2024). 10.1016/j.ultsonch.2024.107079 PMC11459584 39342895

[14] Roobab U , Aadil RM , Kurup SS and Maqsood S , Comparative evaluation of ultrasound‐assisted extraction with other green extraction methods for sustainable recycling and processing of date palm bioresources and by‐products: a review of recent research. Ultrason Sonochem 114:107252 (2025). 10.1016/j.ultsonch.2025.107252 PMC11904522 39985822

[15] Siddiqui SA , Ali Redha A , Salauddin M , Harahap IA and Rupasinghe HPV , Factors affecting the extraction of (poly)phenols from natural resources using deep eutectic solvents combined with ultrasound‐assisted extraction. Crit Rev Anal Chem 55:139–160 (2025). 10.1080/10408347.2023.2266846 37850880

[16] Mazza KEL , Santiago MCPA , Nascimento LSM , Godoy RLO , Souza EF , Brígida AIS *et al*., Syrah grape skin valorisation using ultrasound‐assisted extraction: phenolic compounds recovery, antioxidant capacity and phenolic profile. Int J Food Sci Technol 54:641–650 (2019).

[17] Nayak A , Bhushan B , Rosales A , Turienzo LR and Cortina JL , Valorisation potential of cabernet grape pomace for the recovery of polyphenols: process intensification, optimisation and study of kinetics. Food Bioprod Process 109:74–85 (2018).

[18] Caldas TW , Mazza KEL , Teles ASC , Mattos GN , Brígida AIS , Conte‐Junior CA *et al*., Phenolic compounds recovery from grape skin using conventional and non‐conventional extraction methods. Ind Crop Prod 111:86–91 (2018).

[19] Baranyi J , Pin C and Ross T , Validating and comparing predictive models. Int J Food Microbiol 48:159–166 (1999). 10.1016/s0168-1605(99)00035-5 10443535

[20] Singleton VL and Rossi JA , Colorimetry of total phenolics with phosphomolybdic‐phosphotungstic acid reagents. Am J Enol Vitic 16:144–158 (1965).

[21] Georgé S , Brat P , Alter P and Amiot MJ , Rapid determination of polyphenols and vitamin C in plant‐derived products. J Agric Food Chem 53:1370–1373 (2005). 10.1021/jf048396b 15740008

[22] Giusti MM and Wrolstad RE , Characterization and measurement of anthocyanins by UV‐visible spectroscopy, in Current Protocols in Food Analytical Chemistry, ed. by Wrolstad RE . John Wiley & Sons, New York (2001).

[23] Re R , Pellegrini N , Proteggente A , Pannala A , Yang M and Rice‐Evans C , Antioxidant activity applying an improved ABTS radical cation decolorization assay. Free Radic Biol Med 26:1231–1237 (1999). 10.1016/s0891-5849(98)00315-3 10381194

[24] Rufino MSM , Alves RE , Brito ES , Morais SM , Sampaio CG , Pérez‐Jiménez J *et al*., Metodologia científica: determinação da atividade antioxidante total em frutas pela captura do radical livre DPPH. Embrapa Agroindústria Tropical, Fortaleza, p. 4 (2007).

[25] Nascimento LSM , Santiago MCPA , Oliveira EMM , Borguini RG , Braga ECO , Martins VC *et al*., Characterization of bioactive compounds in Eugenia brasiliensis lam. (Grumixama). Nutr food technol open Access 3:1–6 (2017). 10.16966/2470-6086.146.

[26] Santiago MCR , Gouvêa ACS , Godoy RLO , Nogueira JN and Rosa JS , Adaptação de um método por cromatografia líquida de alta eficiência para análise de antocianinas em suco de açaí (Euterpe oleracea Mart.). Embrapa Agroindústria de Alimentos, Rio de Janeiro (2010).

[27] Mitic M , Jankovic S , Mitic S , Kocic G , Maskovic P and Dukic D , Optimization and kinetic modelling of total phenols and flavonoids extraction from Tilia cordata M. Flowers. S Afr J Chem 75:64–72 (2021).

[28] Rocha CB and Noreña CPZ , Microwave‐assisted extraction and ultrasound‐assisted extraction of bioactive compounds from grape pomace. Int J Food Eng 16:1–10 (2020).

[29] Goula AM , Thymiatis K and Kaderides K , Valorization of grape pomace: drying behavior and ultrasound extraction of phenolics. Food Bioprod Process 100:132–144 (2016).

[30] Luque‐Alcaraz AG , Hernández‐Téllez CN , Graciano‐Verdugo AZ , Toledo‐Guillén AR and Hernández‐Abril PA , Exploring antioxidant potential and phenolic compound extraction from *Vitis vinifera* L. using ultrasound‐assisted extraction. Green Process Synth 13:20230258 (2024).

[31] Drevelegka I and Goula AM , Recovery of grape pomace phenolic compounds through optimized extraction and adsorption processes. Chem Eng Process Process Intensif 149:107845 (2020).

[32] Beres C , Costa GNS , Cabezudo I , Silva‐James NK , Teles ASC , Cruz APG *et al*., Towards integral utilization of grape pomace from winemaking process: a review. Waste Manag 68:581–594 (2017). 10.1016/j.wasman.2017.07.017 28734610

[33] Rockenbach II , Rodrigues E , Gonzaga LV , Caliari V , Genovese MI , Gonçalves AE d SS *et al*., Phenolic compounds content and antioxidant activity in pomace from selected red grapes (*Vitis vinifera* L. and *Vitis labrusca* L.) widely produced in Brazil. Food Chem 127:174–179 (2011).

[34] Moutinho J , Gouvinhas I , Domínguez‐Perles R and Barros A , Optimization of the extraction methodology of grape pomace polyphenols for food applications. Molecules 28:3888 (2023). 10.3390/molecules28093885 PMC10180386 37175294

[35] González‐Centeno MR , Comas‐Serra F , Femenia A , Rosselló C and Simal S , Effect of power ultrasound application on aqueous extraction of phenolic compounds and antioxidant capacity from grape pomace (*Vitis vinifera* L.): experimental kinetics and modeling. Ultrason Sonochem 22:506–514 (2015). 10.1016/j.ultsonch.2014.05.027 24970116

[36] Spinei M and Oroian M , Microwave‐assisted extraction of pectin from grape pomace. Sci Rep 12:12722 (2022). 10.1038/s41598-022-16858-0 PMC9325980 35882905

[37] Llorent‐Martínez EJ , Ortega‐Barrales P , Zengin G , Mocan A , Simirgiotis MJ , Ceylan R *et al*., Evaluation of antioxidant potential, enzyme inhibition activity and phenolic profile of Lathyrus cicera and Lathyrus digitatus: potential sources of bioactive compounds for the food industry. Food Chem Toxicol 107:609–619 (2017). 10.1016/j.fct.2017.03.002 28263865

[38] Kashyap P , Riar CS and Jindal N , Polyphenol bio‐accessibility and antioxidant activity of in vitro digested ultrasound‐assisted Meghalayan cherry (*Prunus nepalensis*) pomace extract. Biomass Convers Biorefinery 13:14071–14085 (2023).

[39] Tumwesigye SK , Montañez JC , Oliveira JC and Sousa‐Gallagher MJ , Novel intact bitter cassava: sustainable development and desirability optimisation of packaging films. Food Bioprocess Technol 9:801–812 (2016).

[40] Zhu MT , Huang YS , Wang YL , Shi T , Zhang LL , Chen Y *et al*., Comparison of (poly)phenolic compounds and antioxidant properties of pomace extracts from kiwi and grape juice. Food Chem 271:425–432 (2019). 10.1016/j.foodchem.2018.07.151 30236697

[41] Abouelenein D , Mustafa AM , Caprioli G , Ricciutelli M , Sagratini G and Vittori S , Phenolic and nutritional profiles, and antioxidant activity of grape pomaces and seeds from Lacrima di morro d'Alba and *Verdicchio varieties* . Food Biosci 53:102808 (2023).

[42] Stanek‐Wandzel N , Zarębska M , Wasilewski T , Hordyjewicz‐Baran Z , Krzyszowska A , Gębura K *et al*., Enhancing phenolic compound recovery from grape pomace residue: synergistic approach of ultrasound‐ and enzyme‐assisted extraction. ACS Omega 10:23129–23138 (2025). 10.1021/acsomega.5c01321 PMC12163761 40521472

[43] Dominguez‐López I , Pérez M and Lamuela‐Raventós RM , Total (poly)phenol analysis by the Folin‐Ciocalteu assay as an anti‐inflammatory biomarker in biological samples. Crit Rev Food Sci Nutr 64:10048–10061 (2024). 10.1080/10408398.2023.2220031 PMC10700652 37283051

[44] Tyagi K , Lui ACW , Zhang S and Peck GM , Folin‐Ciocalteu, RP‐HPLC, and LC‐MS provide complementary information for describing cider (malus spp.) apple juice. J Food Compos Anal 137:106844 (2025).

[45] Faur CA , Ola D , Bunea CI , Călugăr A , Bora FD , Dulf F *et al*., Characterization of anthocyanins and lipid components in berries of nine red grapevine cultivars. Agronomy 15:111 (2025).

[46] Favre G , Hermosín‐Gutiérrez I , Piccardo D , Gómez‐Alonso S and González‐Neves G , Selectivity of pigments extraction from grapes and their partial retention in the pomace during red winemaking. Food Chem 277:391–397 (2019). 10.1016/j.foodchem.2018.10.085 30502162

